# Neural substrates underlying effortful control deficit in autism spectrum disorder: a meta-analysis of fMRI studies

**DOI:** 10.1038/s41598-022-25051-2

**Published:** 2022-11-29

**Authors:** Karthikeyan Krishnamurthy, Melody M. Y. Chan, Yvonne M. Y. Han

**Affiliations:** 1 Department of Rehabilitation Sciences, The Hong Kong Polytechnic University, Hung Hom, Kowloon, Hong Kong SAR, China; 2 Brain & Cognitive Behaviour Research Foundation, Chennai, India

**Keywords:** Cognitive neuroscience, Development of the nervous system

## Abstract

Effortful control comprises attentional control, inhibitory control, and cognitive flexibility subprocesses. Effortful control is impaired in individuals with autism spectrum disorder, yet its neural underpinnings remain elusive. By conducting a coordinate-based meta-analysis, this study compared the brain activation patterns between autism and typically developing individuals and examined the effect of age on brain activation in each effortful control subprocesses. Meta-analytic results from 22 studies revealed that, individuals with autism showed hypoactivation in the default mode network for tasks tapping inhibitory control functioning (threshold-free cluster enhancement p < 0.001). When these individuals perform tasks tapping attentional control and cognitive flexibility, they exhibited aberrant activation in various brain networks including default mode network, dorsal attention, frontoparietal, visual and somatomotor networks (uncorrected ps < 0.005). Meta-regression analyses revealed that brain regions within the default mode network showed a significant decreasing trend in activation with increasing age (uncorrected p < 0.05). In summary, individuals with autism showed aberrant activation patterns across multiple brain functional networks during all cognitive tasks supporting effortful control, with some regions showing a decrease in activation with increasing age.

## Introduction

Autism spectrum disorder (ASD) is a highly prevalent neurodevelopmental disorder. The prevalence of ASD is estimated to be 1 in 54^1^. Children and adolescents with ASD are characterized by sociocommunicative dysfunction and restricted, repetitive behaviors^2^. For example, these individuals present with inflexible, stereotyped behaviors accompanied by temper outbursts over trivial environmental changes, which often cause a great reduction in their quality of life^3^, as well as considerable emotional stress on their caregivers and the community^4^. Previous research has suggested that these behavioral manifestations are underpinned by impairments in self-regulatory processes^5^, among which deficits in effortful control (EC) have consistently been shown to be one of the processes that play a detrimental role in ASD symptomatology^6–8^.

EC is defined as a top-down, proactive self-regulatory process that enables a person “to inhibit a dominant response in order to perform a subdominant response”^9^. EC involves three subcomponents^10–12^, including the ability to focus attention without being distracted by external stimuli (i.e. attentional control), to inhibit undesirable behaviors (i.e. response inhibition) and to switch between the activation and inhibition of thoughts and behaviors according to different environmental demands (i.e. cognitive flexibility). Previous functional magnetic resonance imaging (fMRI) studies in typically developing (TD) individuals have collectively shown that the activation of multiple brain regions embedded in different brain functional networks contribute to distinct cognitive and perceptual functions^13^. Namely, the frontoparietal network is associated with cognitive control^14^, the salience network is associated with attentional control^15^ and the default mode network is associated with the coordination of other functional networks to support efficient information processing^16^, and together, they are necessary to support the functioning of EC subcomponents. For instance, using the attention network task (ANT)/flanker task to tap the functioning of attentional control, defined as one’s behavioral response from numerous available options under conflicting circumstances, an extensive body of research has shown that the frontal (i.e. frontal eye field^17^, anterior cingulate cortex and lateral prefrontal cortex^18^ and parietal regions (i.e. superior parietal lobe, temporal parietal junction^17^ were recruited. Regarding response inhibition, which is usually tapped by the stop-signal task and Go/No-Go task^19^, a neural circuit comprising the presupplementary motor area, left fusiform gyrus, right dorsolateral prefrontal, and inferior parietal circuits is activated^20^. For cognitive flexibility, which is often measured by the Wisconsin card sorting test (WCST), intraextra dimensional set-shift and reversal learning tasks^21–23^, inferior frontal junction, posterior parietal, and frontopolar cortices are shown to be activated in TD individuals^24^.

Given that ASD individuals have been shown to exhibit EC deficits, it is reasonable to postulate that their brain activation patterns during EC subcomponents might be altered compared to their TD counterparts. Indeed, previous studies have shown that people with ASD exhibit aberrant activation patterns when they perform tasks that tap on attentional control, response inhibition and cognitive flexibility, yet the results remain inconsistent. For instance, when ASD individuals perform attentional control tasks, some researchers reported hypoactivation in multiple brain regions, including the anterior cingulate cortex, midfrontal gyrus, right inferior frontal gyrus and bilateral intraparietal sulcus^25,26^, while others reported hyperactivation in the bilateral frontal gyri. When performing inhibitory control tasks, while Schmitz, Rubia^27^ revealed that the left inferior gyrus and orbitofrontal gyrus were hyperactivated in ASD, Shafritz, Bregman^28^ reported that the ventral prefrontal cortex was instead hypoactivated. Similar inconsistencies were noted when individuals with ASD engaged in tasks tapping cognitive flexibility. While Uddin^23^ reported that the abnormal brain activation in ASD is widely distributed throughout cortical (e.g. dorsolateral prefrontal cortex, anterior cingulate cortex, intraparietal sulcus) and subcortical (e.g. basal ganglia, ventral striatum) brain regions, Yerys, Antezana^29^ revealed abnormal activations specifically in the frontal brain regions. The inconsistencies in these results might be attributed to the heterogeneity across studies in the participants’ demographic backgrounds and study designs. Specifically, previous studies have shown that brain activation patterns during the performance of attentional control^30,31^, response inhibition^32,33^ and cognitive flexibility^34–36^ tasks might be impacted by developmental trajectories, suggesting that controlling for age is necessary to reduce error variance in brain activation during EC tasks. In addition, the presence of emotional stimuli, in contrast to stimuli without emotional components, in EC component tasks might influence brain activation patterns as an effect of the emotion recognition difficulty in autism^37^. Therefore, analyses controlling for the effects of differential stimuli on brain activations across studies are essential for yielding a more comprehensive understanding of neural substrates underlying EC deficits in ASD.

In view of the elusive results and limitations reviewed above, a neuroimaging meta-analysis was planned to examine the brain activation patterns underlying EC deficits in autism. Specifically, seed-based d mapping with permutation of subject images (SDM-PSI^38^, a coordinate-based neuroimaging meta-analysis software, was utilized, as it enabled meta-regression and covariate analyses for which the effects of heterogeneity across studies contributing to inconsistencies in the results could be explored and controlled. Furthermore, given that previous studies have collectively shown that the activation of multiple brain regions embedded in different brain functional networks^39^ are required to support the functioning of EC subcomponents and that individuals with ASD have been shown to manifest abnormal brain network functioning^40–42^ that contributes to self-dysregulation^43^, we would like to further explore the brain network correlates of EC deficits in ASD through the registration of consistently coactivated brain regions identified across studies on a standardized brain atlas by means of SDM-PSI^44,45^.

## Methods

### Study design and literature search

This meta-analysis was performed according to the Preferred Reported Items for Systematic Reviews and Meta-Analyses (PRISMA) guideline (Table S1; Moher, Liberati^46^. Relevant studies were searched through electronic databases including the Allied and complementary Medicine Database (AMED), Medline (EbscoHost), PsycINFO (ProQuest), PubMed, Scopus, and Web of Science with a Boolean search using the following keyword combinations [“autism” OR “autism spectrum disorder” OR “asd”] AND [“effortful control” OR “temperament” OR “cognitive” OR “cognitive control” OR “hot executive function”] AND [“fMRI” OR “functional magnetic resonance imaging” AND “brain activation” OR “brain connectivity”]. A literature search was also conducted in the NeuroSynth database by referring to the terms “asd”, “autism”, “cognitive control”, “effortful”, “executive control”, “inhibitory control” and “cognitive flexibility”. The same terms were also typed in the search bar to look for potential studies. Additionally, published meta-analyses in the BrainMap database were searched, and the reference sections of potential articles were further checked manually. The literature search was conducted twice, i.e. in October 2020 and May 2021, without specifying the publication timeline to confirm that the included datasets in this meta-analysis reflected the current literature.

### Inclusion and exclusion of studies

The retrieved articles were screened for duplicate removal, title screening, abstract screening, and full-text screening processes. Whole-brain fMRI studies on EC-related tasks compared in individuals with ASD and neurotypical controls were included in this meta-analysis. Studies without whole-brain fMRI activation during EC-related tasks, without ASD and control groups, without reporting brain activation in the standard spatial coordinates (MNI or Talairach), animal studies, reviews, meta-analyses, book chapters, commentaries, conference abstracts, resting-state brain activation, and regions of interest-based activation were excluded. The included studies were then screened for EC-related experiments addressing attention, inhibitory control, and cognitive flexibility subcomponents. Studies presented with more than one eligible experimental result in an EC subcomponent were pooled within the respective subcomponent. All screening processes were conducted independently by the first and second authors with the accompanying decisions recorded in the Endnote reference software and Excel spreadsheet. Any discrepancies were resolved by consulting with the third author and reached a consensus before finalization.

### Data extraction and recoding

The first author extracted the demographics, experimental procedures, and fMRI details from the included papers and entered them into the database. The second author validated the entries to maximize accuracy. The demographic data comprised the sample size, mean age, mean intelligence, the number of female and male participants in both groups and their matching criteria. The mean age was further grouped into three categories: children (4–11.11 years), adolescents (12–17.11 years), and adults (above 18 years). Experimental procedures included information about the task with stimulus presentation and the type of baseline comparison. The brain activation for the typical and ASD groups during EC-related tasks was categorized into neutral (presence of unanimated stimuli) and socioemotional (presence of animated stimuli) components.

### Data analysis

The meta-analysis between the two groups during EC components (attention, inhibitory control, and cognitive flexibility) was conducted separately using a random-effects model at two levels, i.e. the main analysis combined both neutral and socioemotional stimuli, and a subgroup analysis that included only neutral stimuli was conducted. The meta-analysis was conducted using seed-based d-mapping – permutation of subject images (SDM-PSI) software (version 6.21), which allows for estimating the population effect size with minimal bias via a subject-level permutation test. The program also enhances true positive effects using the familywise error correction method derived from threshold-free cluster enhancement (TFCE) statistics. Furthermore, the algorithm supports meta-regression analysis using a study-level permutation test on the given moderators^38,47^. The analysis began with preprocessing of data on each EC component separately with anisotropy = 1, isotropic full width at half maximum (FWHM) = 20 mm, voxel size = 2 mm on the gray matter mask. Subsequently, the mean was estimated of each EC component by deducting the activation map of the ASD group from the TD group, i.e. (ASD-TD) contrast. Finally, to understand how age modulates the abnormal brain activation in ASD, meta-regression was conducted, for brain clusters that showed significant between-group differences, using a simple linear regression model weighted as the square root of the sample size and limited to predictions within the SDM cutoff values (− 1 to 1)^48^. This analysis revealed the brain regions with significant correlations between the changes in hypoactivation/hyperactivation and chronological age in the ASD group relative to the TD group, with an assumption that the age effect on TD is constant. Regarding the significance threshold, we first identify significant clusters with the threshold p = 0.005 (uncorrected), Z > 1 and a cluster size (k) larger than 10 voxels, given this has been suggested to be effective in controlling type I error while maintaining adequate sensitivity^49,50^. We further verify the results with the threshold p = 0.05 (TFCE-corrected), Z > 1, k > 10, using the pipeline suggested by Albajes-Eizagirre et al.^38^. As the meta-regression is exploratory in nature, the significance level was kept at p < 0.05 (uncorrected), Z > 1 and k > 10. Additionally, the significant brain clusters obtained from the meta-analysis were further parcellated under corresponding brain networks using “freesurfer” software^39,49^. The heterogeneity of the included studies in the meta-analysis was assessed using I-squared (I^2^) statistics, with low, medium, and high heterogeneity determined with the respective values of 25%, 50% and 75%^50,51^. To assess the risk of publication bias, a funnel plot across studies was conducted to ascertain linkages between the calculated and study effect sizes that were greater than chance^52^. In cases of publication bias, the funnel plot appears symmetrical at the top, and data points are missed from the middle and bottom sections of the plot. Subsequently, Egger’s tests on the peak coordinates demonstrate a dissociation between the ASD and control groups during EC component tasks. A significant Egger’s value denotes a small study effect. However, occasionally smaller studies may yield larger effects than studies with larger sample sizes, and this phenomenon occurs as the result of publication bias.

## Results

### Study selection

**Figure 1:**
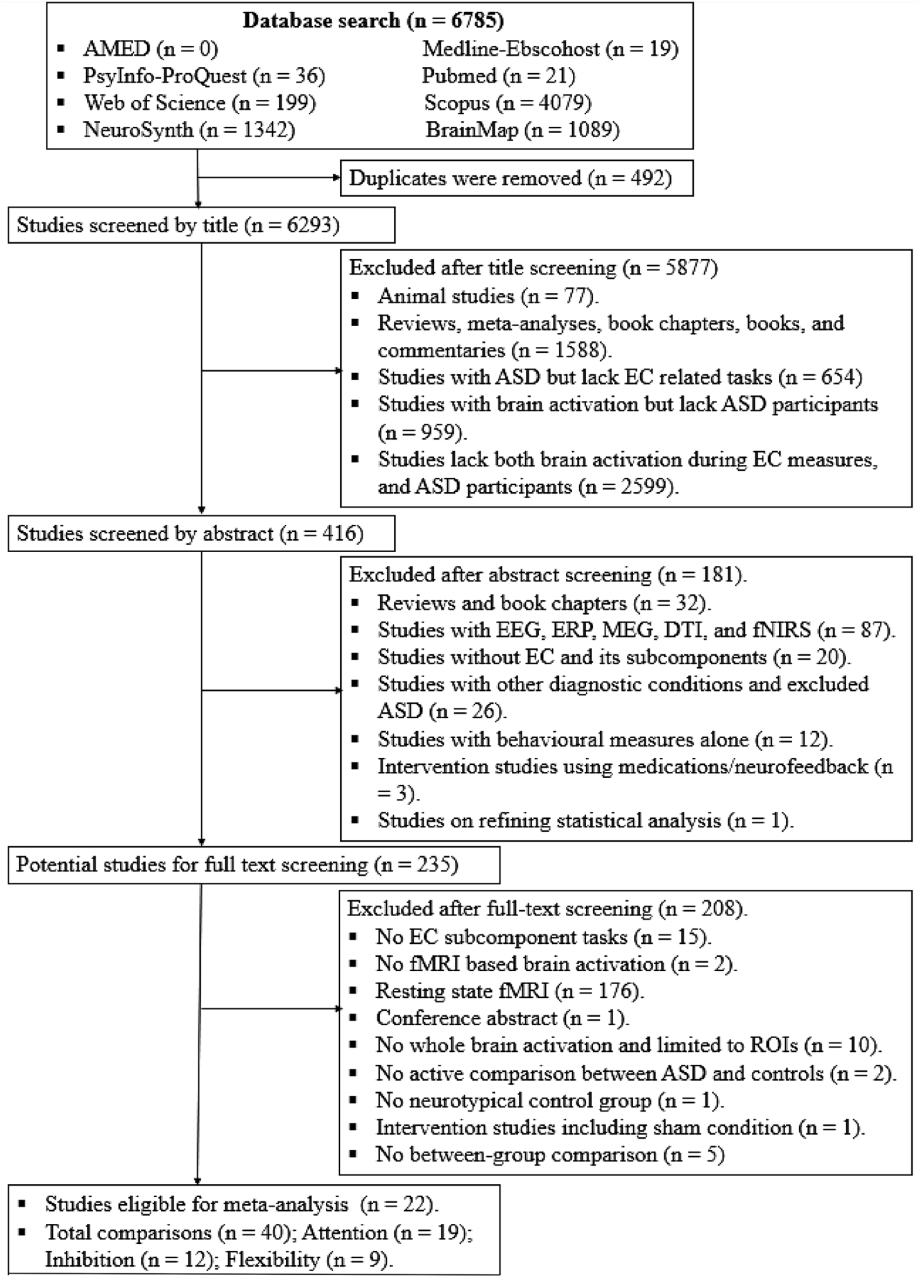
PRISMA flowchart of screening studies.

A sum of 6785 records were obtained from the eight electronic databases. After removing 492 duplicated records, the titles of 6293 records were screened. With exclusion criteria being applied, 5877 records were excluded, while 416 records remained for abstract screening. While 181 records were excluded during abstract screening, the remaining 235 studies were remained for full-text screening. Twenty-two studies (including 40 comparisons) were finally included in the meta-analysis. The article screening process is outlined in Fig. 1.

### Attentional control

#### Study characteristics

Fourteen studies containing 19 comparisons were included in the meta-analysis, which compared 266 individuals with ASD (48 children, 108 adolescents, and 110 adults) with 297 healthy controls (53 children, 127 adolescents, and 117 adults). The demographic and experimental details of the included studies are summarized in Table 1.

**Table 1:** fMRI studies (22 studies; 40 experiments) included in the meta-analysis.

Study	Demographic data					Experimental design						
	Sub-groups	Sample size—ASD: HC	Mean IQ(SD)-ASD:HC	Sex (F; M) ASD: HC	ASD severity measures	Symptom severity score: M (SD)—ASD: HC	Other measures	Subject matching criteria	Task and stimuli presentation (with neutral or socio-emotional component)	EC components—Attentional control/inhibitory control/cognitive flexibility (experimental condition)	Baseline	
Dcruz, 2016	ASD-HC	17:23	103.90 (15.50): 110.90 (9.90)	5;12: 5;18	ADI-R	2.5 (1.4): NA (NA)	RBS-R	Age, gender, IQ	Reversal learning task: 2 and 4 choice—(Neutral stimuli)	Cognitive flexibility (4-choice reversal)	Blank screen	
Dirks, 2020	ASD-HC	24:33	101.5 (18.25): 108.38 (11.92)	3;21: 11;22	ADOS-2nd edition	10.91 (3.23)	BRIEF-2; RBS-R; SCQ	Age and IQ	Set-shifting task—(Neutral stimuli)	Cognitive flexibility (mixed > color + shape; mixed > color blocks)	Blocks of low-level fixation	
Duerden, 2013	ASD-HC	16:17	111.89 (13.71): 114.32 (14.8)	5;11: 5;12	ADI-R; ADOS-G	No total score	NA	IQ	Emotional Go/NoGo task—(Socio-emotional stimuli)	Inhibitory control (NoGo > go)	Cue fixation	
Fan, 2012	ASD-HC	12:12	115 (14):120 (15)	3;9: 2;10	ADI-R; ADOS-G	ADI-R—38.4 (13.4); ADOS-G—12.2 (4.1)	NA	Age, IQ, gender, and handedness score	Attention Network Test—(Neutral stimuli)	Attentional control (flanker conflict; alert by conflict; and no cue > double cue)	Cue fixation cross	
										Inhibitory control (disengaging)		
Gilbert, 2008	ASD-HC (only right handers)	15:18	119 (14):119 (11)	3;12: 5;13	ADI-R; ADOS-G	No total score	NA	Age, and IQ	Alphabet task—(Neutral stimuli)	Cognitive flexibility (stimulus orientation > stimulus; stimulus orientation < stimulus)	Classify straight or curved pattern from non-alphanumeric non-meaningful stimuli	
Gilbert, 2009	ASD-HC (only right handers)	16:16	Full scale IQ—NA	2;14: 4;12	ADOS-G	No total score	NA	Age, IQ (verbal and performance)	Alphabet task—(Neutral stimuli)	Cognitive flexibility (mentalizing > non-metalizing)	Non-mentalizing and stimulus orientation stimuli	
Gordon, 2020	ASD-HC	64:77	103.72 (12.88): 110.05 (11.23)	11;53: 16;61	ADOS	No total score	NA	Age, and gender	Rapid Preparing to Overcome Prepotency Task—(Neutral Stimuli)	Attentional control (red, and green cues) Inhibitory control (red probe)	Fixation cross	
Hames, 2016	ASD-HC	6:6	NA	2;4: 2;4	ADOS	NA	NA	NA	The modified child ANT task—(Neutral stimuli)	Attentional control (incongruent > congruent)	Fixation cross	
Kana, 2007	ASD-HC	12:12	110.1 (12.6): 117.0 (8.7)	1;11: 1;11	ADI-R; ADOS	NA	NA	Age, and IQ	The response inhibition task—(Neutral stimuli)	Attentional control (No-go); Inhibitory control (1-back inhibition)	Press every letter except ‘A’	
Kathleen, 2012	ASD-HC	14:14	113.21 (NA): 116.64 (NA)	2;12: 3;11	ADI-R; ADOS-G	NA	BRIEF-2; RBS-R; ASI (NEPSY-II); TEA-Ch; TMT	Age, gender, and IQ	Set-shifting task—(Neutral stimuli)	Cognitive flexibility (extra > intra-dimension)	Fixation cross	
										Attentional control (extra-dimension > fixation)		
Keehn, 2016	ASD-HC	16:21	Verbal = 112 (17):106 (10); Non-verbal = 112 (14):107 (11)	2;14: 5;16	ADI-R; ADOS	No total score	NA	Age, nonverbal IQ, and motion during MRI scanning	Rapid serial visual presentation—(Neutral stimuli)	Inhibitory control (target color > non-target color)	Number task	
										Attentional control (target present neutral)		
Murphy, 2017	ASD-HC	23:35	114.90 (16.30): 121.97 (10.63)	6;17: 18;17	ADI-R; ADOS-G	ADOS (overall severity): 6.61 (2.28)	NA	Age, and IQ	Non-social dot probe task—(Neutral stimuli)	Attentional control (neutral 18 < > bias 18; neutral 18 < > bias 72)	Fixation cross	
Ohta, 2012	ASD-HC	24:25	112.80 (6.40): 109.20 (7.70)	3;21: 3;22	AQ	AQ = 36.1 (5.9): 15.6 (7.4)	HADS	Age, and IQ	Rapid serial visual presentation—(Neutral stimuli)	Attentional control (distractor present vs. absent)	Fixation cross	
Sabatino, 2013	ASD-HC	15:17	109.9 (20.3): 127.0 (8.1)	2;13: 5;12	ADOS; AQ	AQ = 24.7 (13.1):12.4 (5.3)	RBS-R; SRS-SR	Age, and gender	Visual oddball target detection task—(Neutral, and socio-emotional stimuli)	Attentional control (face, and non-face stimuli)	Fixation cross	
Schmitz, 2006	ASD-HC	10:12	105 (14): 106 (13)	0;10: 0;12	ADI-R	No total score	NA	Age, and IQ	Three tasks, (a) Go/NoGo task, (b) stroop task, (c) switch task—(Neutral stimuli)	Inhibitory control (correct NoGo; correct stroop)	Fixation cross	
										Cognitive flexibility (correct switch)		
Shafritz, 2015	ASD-HC	15:15	101.5 (18.6): 115.2 (9.3)	3;12: 3;12	ADI-R; ADOS-G	NA	NA	NA	Block design Go/NoGo task—(Neutral and socio-emotional stimuli)	Inhibitory control (‘x’ NoGo > letter NoGo)	Fixation cross	
										Attentional control (emotion NoGo > letter NoGo)		
Solomon, 2009	ASD-HC (only right handers)	22:23	107 (14): 113 (11)	5;17: 5;18	ADOS-G	No total score	SCQ	NA	Preparing to Overcome Prepotency (POP) task—(Neutral stimuli)	Cognitive flexibility (red > green)	Fixation cross	
										Attentional control (red > baseline)		
Takarae, 2007	ASD-HC	13:14	105.90 (12.30): 110.30 (13.70)	NA	ADOS-G	NA	NA	Age, and IQ	Visually guided saccade task—(Neutral stimuli)	Attentional control (saccadic target movement right or left)	Fixation cross	
Thakkar, 2008	ASD-HC	12:14	No full scale IQ	2;10: 6;8	ADI-R; ADOS	NA	NA	Age, sex, and handedness	Saccadic paradigm—(Neutral stimuli)	Inhibitory control (correct prosaccade + antisaccade vs. fixation)	Fixation cross	
Vaidya, 2011	ASD-HC	11:14	113.85 (15.40): 119.17 (14.19)	3;8: 3:11	ADI-R; ADOS	NA	NA	Age, and IQ	Arrow and Gaze tasks—(Stroop like task; neutral, and socio-emotional stimuli)	Attentional control (congruent > neutral)	Fixation cross	
										Inhibitory control (incongruent > congruent)		
Velasquez, 2017	ASD-HC	19:22	115.53 (12.82): 112.27 (11.84)	6;13: 6;16	ADI-R; ADOS	ADI-R = No total score. ADOS = 3.77 (2.21)	SADS; whole brain activation	Age, IQ, and gender	Go/NoGo task—(Neutral and socio-emotional stimuli)	Inhibitory control (letter/face NoGo > Go)	Fixation cross	
										Attentional control (face NoGo > letter NoGo)		
Yerys, 2015	ASD-HC	20:19	114.70 (14.50): 119.58 (13.25)	4;16: 6;13	ADI-R; ADOS	ADI-R = No total score. ADOS = 11.15 (2.92)	NA; whole brain activation	Age, IQ, and gender	The set-shifting task—(Neutral stimuli)	Cognitive flexibility (switch > stay)	Stay/Switch instructions in the centre of the screen	

*ASI (NEPSY-II)* animal sorting and inhibition subtests from NEPSY-II, *BRIEF* behaviour rating inventory of executive function, *SCQ* social communication questionnaire, *DISC-IV* diagnostic interview schedule for children, *DAWBA* development and well-being assessment, *RBS-R* repetitive behaviour scale—revised questionnaire, *DISCO* diagnostic interview for social and communication disorders, *HADS* hamilton anxiety and depression scale, *SADS* social avoidance and distress scale, *TEA-Ch* test of everyday attention for children, *TMT* trail making test.

#### Brain activation

**Figure 2:**
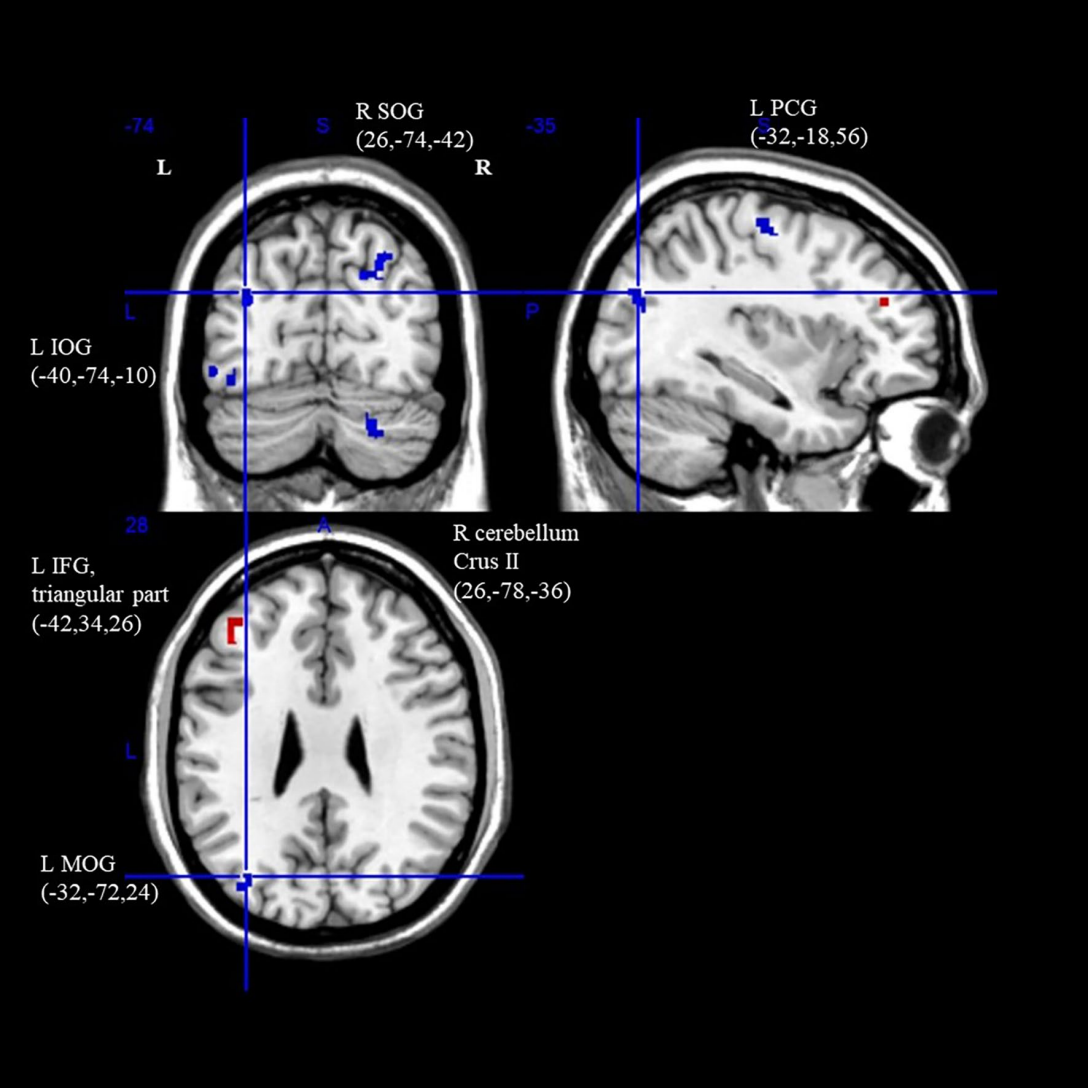
Differences in brain activation between ASD and HC during attentional control. Cluster with red and blue colours indicates hyperactivation and hypoactivation when compared with HC (*p* < 0.005, uncorrected; *L* left, *R* right, *SOG* superior occipital gyrus, *MOG* middle occipital gyrus, *IOG* inferior occipital gyrus, *IFG* inferior frontal gyrus, *PCG* precentral gyrus).

As shown in Table 2 and Fig. 2, the ASD group showed significantly greater activation in the frontoparietal network (FPN) than the TDC group. Simultaneously, there were also significant deactivations in the visual network (VN), FPN and somatomotor network (SMN), with mean age as a covariate (uncorrected *ps* < 0.005). For the FPN, an activation peak was observed in the left frontal region (triangular part), where the cluster extended from the mid to inferior frontal region. In contrast, the deactivation peak of FPN was observed in the right cerebellum crus II, where the cluster extended from the right cerebellum crus I and II to its hemispheric lobule VI. For SMN, the deactivation peak was observed in the left precentral gyrus. The clusters of these peaks were restricted to the corresponding brain regions alone. For VN, the peak was observed in the left inferior occipital gyrus, and the cluster extended from the peak site to the left fusiform gyrus and fasciculus. These clusters did not survive TFCE-corrected p = 0.05 threshold. The I^2^ statistic for the left inferior frontal gyrus (triangular part; 6.98%), right cerebellum crus II (7.59%), right superior occipital gyrus (18.12%), and indicated low heterogeneity.

**Table 2:** fMRI meta-analysis on EC components (attention) with age as a covariate.

Brain regions with significant peak activation						Cluster breakdown	Network parcellation
Anatomical region	ASD > TD/ASD < TD	Total voxels	MNI coordinates	SDM-Z	*p (uncorrected)*	Anatomical regions (Broadmann areas)	
Left inferior frontal gyrus, triangular part	ASD > TD	47	− 42,34,26	3.580	< 0.0005	Left middle frontal gyrus (BA45, 46)	FPN
						Left inferior frontal gyrus, triangular part (BA45, 46)	
						Corpus callosum	
						Right anterior thalamic projections (NA)	
Right cerebellum, crus II	ASD < TD	167	26,− 78,− 36	− 3.465	< 0.0005	Right cerebellum, crus I and II	FPN
						Right cerebellum, hemispheric lobule VI (BA19, 37)	
Right superior occipital gyrus	ASD < TD	135	26,− 74,42	− 3.253	< 0.0005	Right superior occipital gyrus (BA7, BA18, BA19)	VN
						Corpus callosum	
						Right cuneus cortex (BA7, BA18, BA19)	
Left inferior occipital gyrus	ASD < TD	51	− 40,− 74,− 10	− 2.967	< 0.005	Left inferior occipital gyrus (BA19, BA37)	VN
						Left inferior temporal gyrus (BA37)	
						Left fusiform gyrus (BA19)	
						Left middle temporal gyrus (BA37)	
						Left inferior network, inferior longitudinal fasciculus (NA)	
Left precentral gyrus	ASD < TD	41	− 32,− 18,56	− 3.169	< 0.005	Left precentral gyrus (BA6, BA4)	SMN
						Corpus callosum	
Left middle occipital gyrus	ASD < TD	31	− 32,− 72,24	− 3.151	< 0.005	Left middle occipital gyrus (BA19, BA39)	VN
						Left inferior network, inferior longitudinal fasciculus (NA)	
						Left superior longitudinal fasciculus I	
Corpus callosum	ASD < TD	13	30,− 34,− 10	− 2.863	< 0.005	Corpus callosum	N/A
						Right hippocampus (BA20, BA37)	
						Right parahippocampal gyrus (BA37)

### Inhibitory control

#### Study characteristics

Ten studies containing 12 comparisons were included in the meta-analysis, which compared 187 individuals with ASD (11 children, 80 adolescents, and 96 adults) with 216 healthy controls (14 children, 98 adolescents, and 104 adults). The demographic and experimental details of the included studies are summarized in Table 1.

#### Brain activation

**Figure 3:**
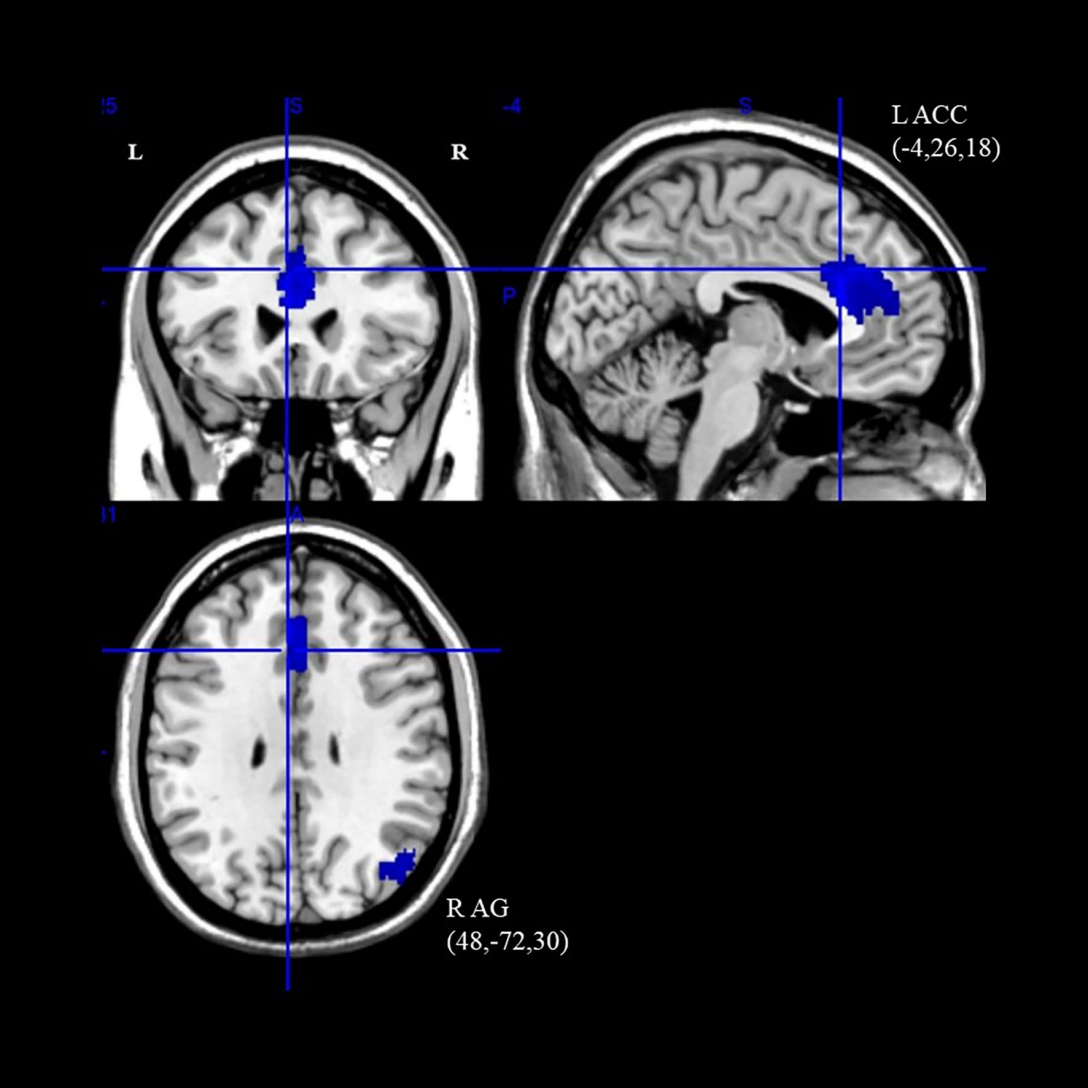
Differences in brain activation between ASD and HC during inhibitory control. Cluster with blue colour indicates hypoactivation when compared with HC (*p* < 0.005, uncorrected; *L* left, *R* right, *ACC* anterior cingulate cortex, *AG* angular gyrus).

As shown in Table 3 and Fig. 3, the ASD group showed significantly reduced activation in the default mode network (DMN), and DAN with mean age was a covariate (uncorrected *ps* < 0.005) when compared to the TDC group. For the DMN, a deactivation peak was observed in the left anterior cingulate/paracingulate gyri region, where the cluster extended from the bilateral anterior cingulate gyri to the bilateral median cingulate gyri. For the DAN, a deactivation peak was observed in the right angular gyrus, where the cluster extended from the right angular gyrus to the right middle occipital and temporal gyri regions. Notably, the DMN cluster survived familywise error correction (773 voxels; SDM-Z = -4.24; TFCE-corrected *p* < 0.001). The I^2^ statistic for the left anterior cingulate/paracingulate gyri (4.65%) and right angular gyrus (12.26%) indicated low heterogeneity.

**Table 3:** fMRI meta-analysis on EC components (inhibitory control) with age as a covariate.

Brain regions with significant peak activation						Cluster breakdown	Network parcellation
Anatomical region	ASD > TD/ASD < TD	Total voxels	MNI coordinates	SDM-Z	*p (uncorrected, unless otherwise specified)*	Anatomical regions (Broadmann areas)	
Left anterior cingulate/paracingulate gyri	ASD < TD	773	− 4,26,18	− 4.240	< 0.0001 (uncorrected); < 0.001 (TFCE-corrected)	Left and right anterior cingulate/paracingulate gyri (BA24, BA32)	DMN
						Left median network	
						Left superior frontal gyrus, medial (BA32)	
						Right, and left median cingulate/paracingulate gyri (BA24, BA32)	
						Corpus callosum	
Right angular gyrus (BA39)	ASD < TD	196	48, − 72,30	− 3.162	< 0.005	Right angular gyrus (BA39, BA19)	DAN
						Right middle occipital gyrus (BA19, BA39)	
						Right middle temporal gyrus (BA39)	
						Right angular gyrus (BA19)

### Cognitive flexibility

#### Study characteristics

Eight studies containing 9 comparisons were included in the meta-analysis, which compared 138 individuals with ASD (58 children, 22 adolescents, and 58 adults) with 158 healthy controls (66 children, 23 adolescents, and 69 adults). The demographic and experimental details of the included studies are summarized in Table 1.

#### Brain activation

**Figure 4:**
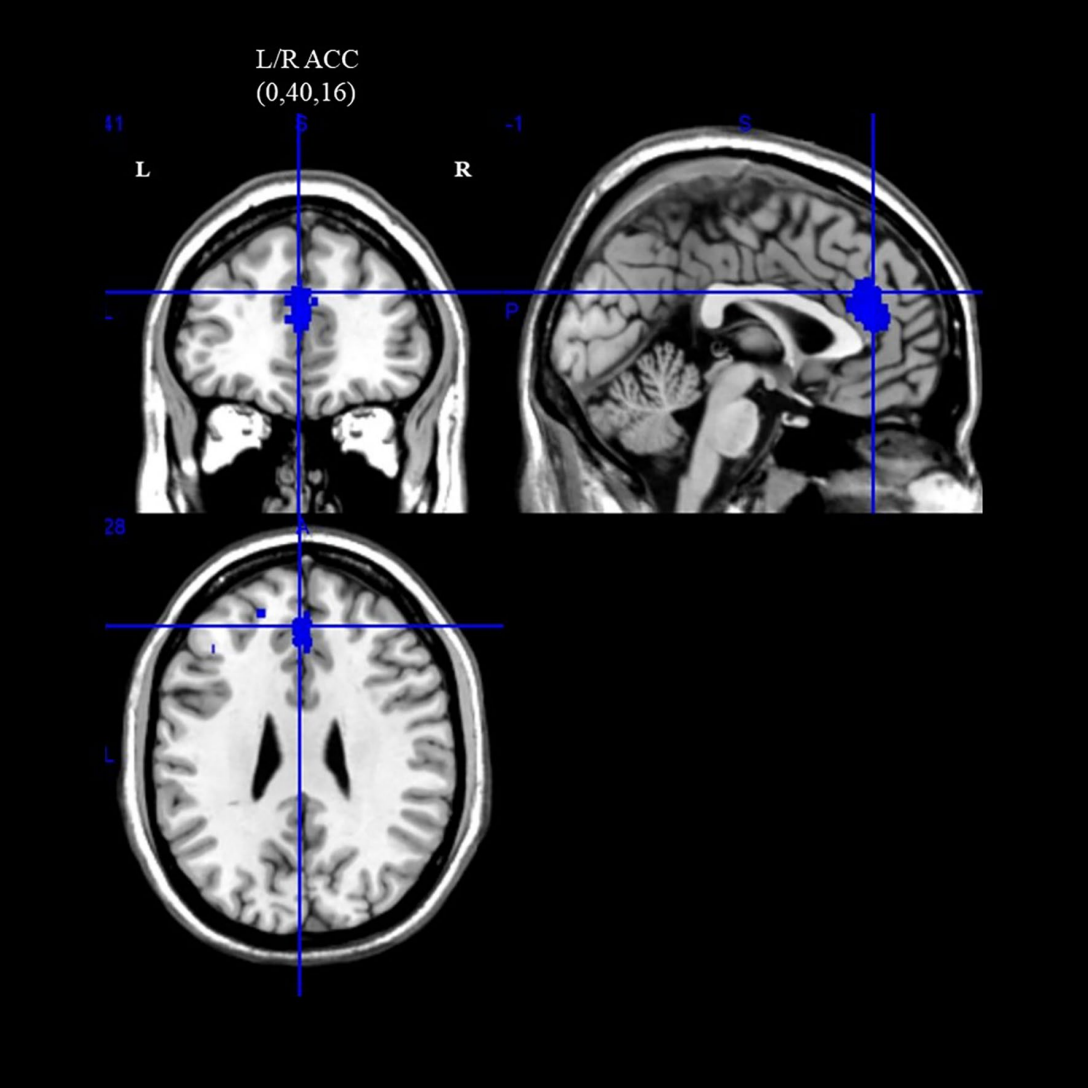
Differences in brain activation between ASD and HC during cognitive flexibility. Cluster with blue colour indicates hypoactivation when compared with HC (*p* < 0.005, uncorrected; *L* left, *R* right, *ACC* anterior cingulate cortex).

As shown in Table 4 and Fig. 4, ASD individuals showed significantly reduced activation in the default mode network (DMN) with mean age were covariates (uncorrected *ps* < 0.005; Fig. 4) when compared to their typically developing counterparts. For the DMN, a deactivation peak was observed in the left anterior cingulate/paracingulate gyri region, where the cluster extended from the bilateral anterior cingulate gyri to the right median cingulate gyri. This cluster did not survive TFCE-corrected p = 0.05 threshold. The I^2^ statistic for the left anterior cingulate/paracingulate gyri (6.23%) indicated low heterogeneity.

**Table 4:** fMRI meta-analysis on EC components (cognitive flexibility) with age as a covariate.

Brain regions with significant peak activation						Cluster breakdown	Network parcellation
Anatomical region	ASD > TD/ASD < TD	Total voxels	MNI coordinates	SDM-Z	*p (uncorrected)*	Anatomical regions (Broadmann areas)	
Left anterior cingulate/paracingulate gyri	ASD < TD	388	0,40,16	− 3.148	< 0.005	Right and left anterior cingulate/paracingulate gyri (BA24,32)	DMN
						Left superior frontal gyrus, medial (BA32)	
						Right median cingulate/paracingulate gyri (BA32)

### Meta-regression

Results of meta-regression of EC components with age as a regressor are shown in Table 5. Regarding attention and inhibitory control tasks, correlations between the changes in brain activation and age for all clusters remained nonsignificant. Regarding cognitive flexibility tasks, the activation of anterior cingulate gyri (MNI coordinates: 0,44,4), of which is was shown to be hypoactivated when compared to TD (Table 4), decreased with increasing age (SDM-Z = − 2.064; p = 0.019).

**Table 5:** Meta-regression of EC components with age as a regressor.

Anatomical region	Decrease/increase in activation with increasing age	Total voxels	MNI coordinates	SDM-Z	*p (uncorrected)*	Anatomical regions (Broadmann areas)	Network parcellation
**EC component: attention**							DMN
*n.s.*							
**EC component: inhibitory control**							
*n.s.*							
**EC component: cognitive flexibility**							
Left anterior cingulate/paracingulate gyri	Decrease	93	0,44,4	− 2.064	0.019	Left anterior cingulate/paracingulate gyri (BA10, BA32)	
						Right anterior cingulate/paracingulate gyri, BA(10)	
						Right superior frontal gyrus, medial (BA10)	

*FPN* fronto-parietal network, *SMN* somato-motor network, *DMN* default mode network, *n.s*. nonsignificant.

### Subgroup analyses with neutral stimuli only

Similar results were obtained when experimental contrasts with socioemotional components were excluded from the analyses for all EC components (Table S2).

### Risk of publication bias

Visual inspection of funnel plots of significant clusters identified in the meta-analyses for attentional control (Fig. S1), response inhibition (Fig. S2) and cognitive flexibility (Fig. S3) showed that the results reported above were not confounded by publication bias. All Egger’s tests were nonsignificant (ps > 0.246), indicating the results were not confounded by small study effects.

## Discussion

This coordinate-based meta-analysis aimed to investigate the neural basis of temperamental EC deficits in individuals with ASD. The literature search yielded 22 fMRI studies with whole-brain data that investigated the brain activation patterns of ASD individuals when they performed tasks tapping EC subprocesses (i.e. attentional control, response inhibition, cognitive flexibility). Our results highlighted two main points. First, when compared to TD individuals, brain functional network deficits underpinning EC were evident. Second, meta-regression analyses revealed that brain regions within the DMN activated during EC show a significant decreasing trend with increasing age.

Consistent with previous findings^53^, our meta-analytic results showed abnormal activation patterns in ASD during all tasks tapping subprocesses underlying EC. Specifically, during attentional control tasks, ASD individuals exhibited aberrant activations in various brain regions within the VN, FPN, and SMN. The aberrant activation of the VN during the attention task is consistent with previous results claiming that individuals with autism demonstrated decreased activation patterns in various occipital regions while detecting visual information, perceiving movements, and processing facial expressions^54^. Deactivation and poor network integration in VN regions while relaying visual information might lead to disrupted visual perceptual abilities in ASD^55^. The left inferior frontal gyrus within the FPN cluster is activated during stimulus-driven attention tasks^56,57^. The hyperactivation of the left inferior frontal gyrus in the ASD group revealed by our meta-analysis might imply that they might recognize high-contrast nontarget stimuli or orienting external cues using contextual information while responding to Attentional control tasks, which warrants further research. As all studies included in our meta-analysis involved attentional control tasks that required participants to provide behavioral responses by pressing buttons, the abnormal activation in the left precentral gyri within the SMN, which is known to be responsible for regulating voluntary motor actions and planning intentional movements of the right extremities^58^, might be associated with the slowness in the response during attention tasks, as shown in previous studies^59^.

During both inhibitory control and cognitive flexibility tasks, ASD individuals exhibited hypoactivation in the left anterior cingulate within the DMN. The anterior cingulate cortex (ACC) has long been implicated as the primary node for monitoring conflicts^60^ and shifting response patterns during inhibitory control^61^ and cognitive flexibility^62^ functions. In this context, the reduced recruitment of the ACC in autism in this meta-analysis was consistent with previous studies showing a significant deactivation and underconnectivity of the ACC during inhibitory control^63^ and cognitive flexibility^23^ subcomponents, which implies that individuals with autism use defective mechanisms during inhibition and flexibility. Along with the DMN, the right angular gyrus within the DAN, a brain network that modulates intentional, target-oriented, top-down endogenous attention^17^, was deactivated during inhibitory control. Activation of the angular gyrus was more pronounced in the healthy population when interference resolution^64^, action withholding, and action cancellation were combined as response inhibition constructs^44^. Therefore, the deactivation of the right angular gyrus in individuals with autism might be explained as a consequence of a difficulty in overcoming prepotent response tendencies either to select appropriate response patterns or to stop the execution of inappropriate responses during task performance.

A previous meta-analysis^65^ has shown that, ASD individuals exhibit aberrant activation patterns in anterior/median cingulate, middle/inferior frontal gyri, inferior parietal lobule, lingual gyrus/cerebellum, and inferior occipital gyrus when compared to TD individuals during cognitive control tasks. Although different constructs were studied, our results were largely consistent with their findings. This is interesting because the definition of cognitive control and EC is fundamentally different. While cognitive control is defined as the ability to adjust behavior flexibly according to changing task demands^66^, EC refers to one’s tendency, influenced by temperaments, to employ top-down control for self-regulation^67^. Yet, it has been recently suggested that EC is essentially equivalent to cognitive control for self-regulation, of which both constructs refers to the basic cognitive processes that support complex cognition^68^. In other words, our meta-analysis, together with Lukito, Norman^65^, collectively provide empirical neuroimaging evidence to support the understanding of EC and cognitive control as constructs that are functionally identical to each other. It is also interesting to note that, although significant group differences were found in all the three subprocesses supporting EC, the effect sizes for the three subprocesses were different. For instance, with a smaller study size for inhibitory control and cognitive flexibility subprocesses, the abnormal brain activation patterns in ASD when compared to TD were statistically more significant, while for attentional control tasks, ASD showed less significant differences when compared to TD in spite of a larger study size. The smaller effect size in attentional control tasks may be a consequence of greater heterogeneity across studies. Although there are indeed studies showing that people with autism have notable deficits in attentional control, especially when they are asked to direct attention to socially-relevant stimuli^69^, other clinical reports revealed that, some ASD individuals have comparable performance in attending to both socially-relevant (e.g. eye gaze^70^) and nonsocial (e.g. flankers^71^) stimuli. In line with these clinical findings, our meta-analytic results suggested that the functioning of brain networks supporting attentional control might vary across ASD individuals, and the EC deficits commonly observed across the majority of ASD individuals could be attributed to deficits in the brain functional networks supporting inhibitory control and cognitive flexibility.

Finally, in line with our expectations, the meta-regression analysis showed that the activation of DMN (left anterior cingulate/paracingulate gyri) during cognitive flexibility tasks has a negative association with age in ASD. A previous study in healthy individuals showed that the magnitude of ACC recruitment decreases with increasing age^72^. Such developmental patterns were consistent with the ACC in terms of deactivation in autism, which could be a result of the cognitive flexibility deficits across age groups.

## Limitations

Although this meta-analysis has provided important insights regarding the neural underpinnings of EC deficits in ASD, some limitations should be noted. First, it included a limited number of studies despite an extensive literature search conducted using various electronic databases and manual search methods. Specifically, we have attempted to retrieve the most comprehensive set of records by using the most common terms (confirmed by the preliminary search conducted in October 2020) seen in the autism and effortful control literature. During the preliminary search, we observed that although some relevant papers did include the use of specific terms (e.g. Asperger’s, Simon task, Stroop task), some generic terms we used in the literature search i.e. “autism” and “cognitive” were seen in these papers along with the specific terms. Therefore, we believe that the current search terms are adequate for capturing the literature we need for this review. Although the chance is minimal, we acknowledged the possibility of missing some relevant papers due to the exclusion of specific search terms. The more important factor contributing to the limited number of studies included is the lack of whole-brain analysis data in some papers. For instance, there were ten studies that were excluded because their analyses were limited to specific regions of interest. If not limited to whole-brain studies, the meta-analysis power would have been higher, and it might have yielded a comprehensive understanding of EC functions in autism. Second, due to the limited number of papers included, we chose a less stringent significance threshold for meta-regression, given the analysis between abnormal brain activation and age was considered exploratory. Cautious interpretation of meta-regression results were warranted, and it is hoped that more longitudinal studies regarding EC subcomponents could be done to help understand the relationship between age and brain activation changes. Third, the age range (in years) of our meta-analysis was limited to 9.58–39.00. The degree of brain regions and accompanying neural network involvement outside this range is still unclear. Hence, it is recommended that forthcoming fMRI studies on EC components confined with this age range be performed to comprehensively ascertain the developmental trajectories of EC functions.

## Conclusion

This coordinate-based fMRI meta-analysis investigated brain activation patterns between individuals with autism and healthy controls during EC subcomponents, including Attentional control, inhibitory control, and cognitive flexibility. The available whole-brain fMRI data in each EC subcomponent were synthesized independently using the SDM-PSI meta-analytic algorithm. In conclusion, the meta-analysis found that the functional brain networks supporting attentional control, inhibitory control, and cognitive flexibility systems are aberrant in autism, and the dysfunctional patterns are moderated by age. These results collectively provide insights regarding the neural correlates of EC deficits in ASD.

## Supplementary Information

Supplementary Figures.

Supplementary Tables.

## Data Availability

The referenced datasets analysed during the current study are available from the corresponding author on reasonable request.
