# Supplementary material for: Neural substrates underlying effortful control deficit in autism spectrum disorder: a meta-analysis of fMRI studies
Source: Sci Rep. 2022 Nov 29;12:20603. doi: 10.1038/s41598-022-25051-2 (PMC9708641; doi:10.1038/s41598-022-25051-2)
Supplement: Supplementary file 1 — Supplementary Figures. [file 41598_2022_25051_MOESM1_ESM.docx]

**Supplementary figure captions**

Figure S1: Funnel plots of the significant peak coordinates during attention tasks with combined stimuli. The x-axis denotes the effect size (Hedges’s g) between two groups, and the y-axis is the precision (1/SE) with random effects model. Visual inspection of the funnel plots did not show obvious asymmetries in the left inferior frontal gyrus (triangular part; Fig. S1a), right cerebellum crus II (Fig. S1b), right superior occipital gyrus (Fig. S1c), left fusiform gyrus (Fig. S1d), left precentral gyrus (Fig. S1e) and the Egger’s test of these brain regions were non-significant (*ps* > 0.856), indicating no publication bias and no prominent small-study effects.

Figure S2: Funnel plots of the significant peak coordinates during inhibitory control tasks with combined stimuli. The x-axis denotes the effect size (Hedges’s g) between two groups, and the y-axis is the precision (1/SE) with random effects model. Visual inspection of the funnel plots did not show obvious asymmetries in the left anterior cingulate/paracingulate gyri (Fig. S2a), right angular gyrus (Fig. S2b). The Egger’s tests of these brain regions were non-significant (*ps* > 0.246).

Figure S3: Funnel plots of the significant peak coordinates during cognitive flexibility tasks with combined stimuli. The x-axis denotes the effect size (Hedges’s g) between two groups, and the y-axis is the precision (1/SE) with random effects model. Visual inspection of the funnel plots did not show obvious asymmetries in the left anterior cingulate/paracingulate gyri. The Egger’s tests of these brain regions were non-significant (*ps* > 0.994), indicating no publication bias or prominent small-study effects of included studies in the meta-analysis.

**Figure S1**

| a) Left inferior frontal gyrus (Triangular part; -42,34,26); Egger’s test *p* = 1.000  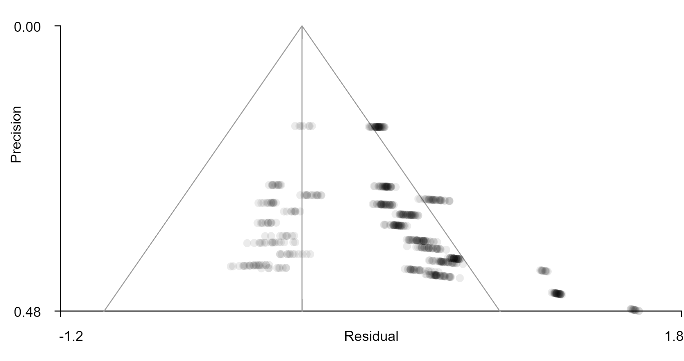 | b) Right cerebellum, crus II (26,-78,-36);  Egger’s test *p* = 0.856  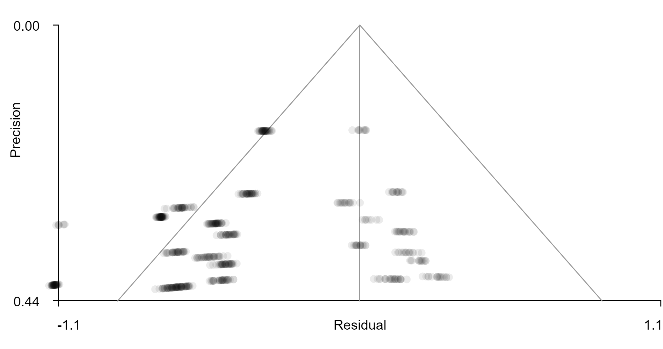 |
| --- | --- |
| c) Right superior occipital gyrus (26,-74,-42);  Egger’s test *p* = 0.995  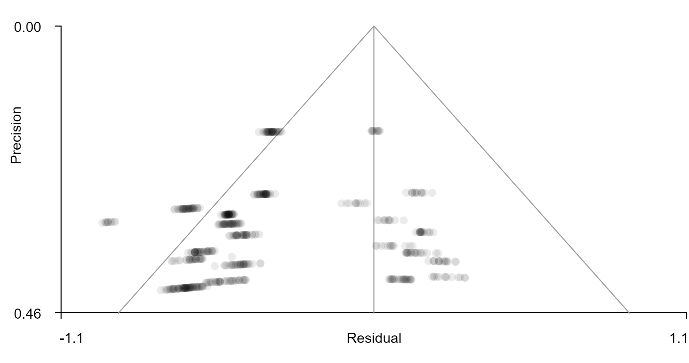 | d) Left fusiform gyrus (-42,-62,-16);  Egger’s test *p* = 0.996  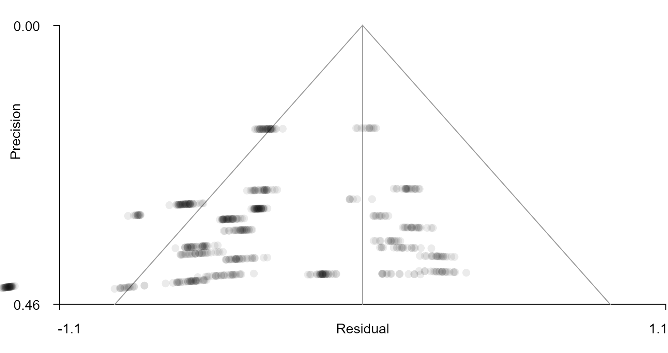 |
| e) Left precentral gyrus (-32,-18,56);  Egger’s test *p* = 0.905  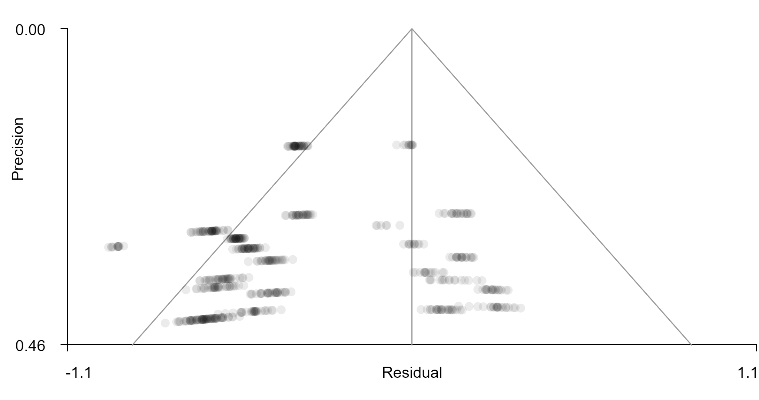 |  |

**Figure S2**

| a) Left anterior cingulate/paracingulate gyri  (-4,26,18); Egger’s test *p* = 0.246  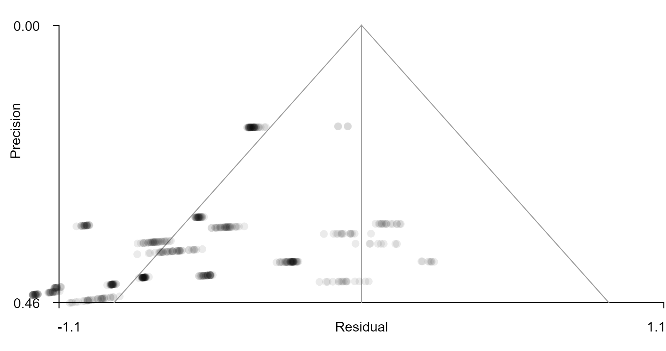 | b) Right angular gyrus (48,-72,30);  Egger’s test *p* = 0.942  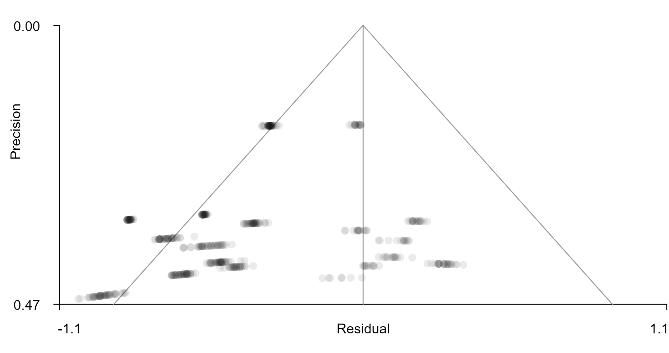 |
| --- | --- |

**Figure S3**

| Left anterior cingulate/paracingulate gyri (0,40,16); Egger’s test *p* = 1.000  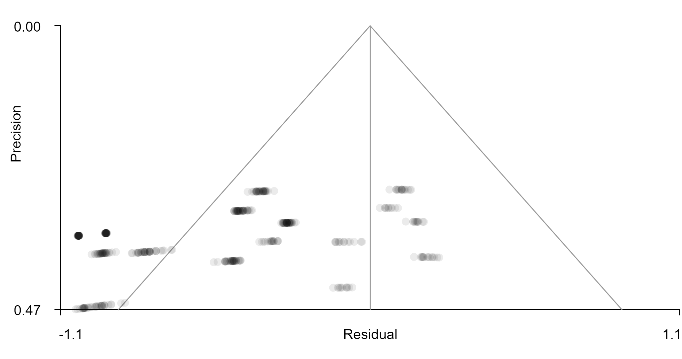 |  |
| --- | --- |
