# Supplementary material for: Neural substrates underlying effortful control deficit in autism spectrum disorder: a meta-analysis of fMRI studies
Source: Sci Rep. 2022 Nov 29;12:20603. doi: 10.1038/s41598-022-25051-2 (PMC9708641; doi:10.1038/s41598-022-25051-2)
Supplement: Supplementary file 2 — Supplementary Tables. [file 41598_2022_25051_MOESM2_ESM.docx]

**Table S1: PRISMA checklist**

| **Section/topic** | **#** | **Checklist item** | **Reported on page #** |
| --- | --- | --- | --- |
| **TITLE** | | |  |
| Title | 1 | Identify the report as a systematic review, meta-analysis, or both. | 1 |
| **ABSTRACT** | | |  |
| Structured summary | 2 | Provide a structured summary including, as applicable: background; objectives; data sources; study eligibility criteria, participants, and interventions; study appraisal and synthesis methods; results; limitations; conclusions and implications of key findings; systematic review registration number. | 2 |
| **INTRODUCTION** | | |  |
| Rationale | 3 | Describe the rationale for the review in the context of what is already known. | 3-5 |
| Objectives | 4 | Provide an explicit statement of questions being addressed with reference to participants, interventions, comparisons, outcomes, and study design (PICOS). | 5-6 |
| **METHODS** | | |  |
| Protocol and registration | 5 | Indicate if a review protocol exists, if and where it can be accessed (e.g., Web address), and, if available, provide registration information including registration number. | 6 |
| Eligibility criteria | 6 | Specify study characteristics (e.g., PICOS, length of follow-up) and report characteristics (e.g., years considered, language, publication status) used as criteria for eligibility, giving rationale. | 6-7 |
| Information sources | 7 | Describe all information sources (e.g., databases with dates of coverage, contact with study authors to identify additional studies) in the search and date last searched. | 6 |
| Search | 8 | Present full electronic search strategy for at least one database, including any limits used, such that it could be repeated. | 6 |
| Study selection | 9 | State the process for selecting studies (i.e., screening, eligibility, included in systematic review, and, if applicable, included in the meta-analysis). | 6-7 |
| Data collection process | 10 | Describe method of data extraction from reports (e.g., piloted forms, independently, in duplicate) and any processes for obtaining and confirming data from investigators. | 7 |
| Data items | 11 | List and define all variables for which data were sought (e.g., PICOS, funding sources) and any assumptions and simplifications made. | 7 |
| Risk of bias in individual studies | 12 | Describe methods used for assessing risk of bias of individual studies (including specification of whether this was done at the study or outcome level), and how this information is to be used in any data synthesis. | NA |
| Summary measures | 13 | State the principal summary measures (e.g., risk ratio, difference in means). | 8-9 |
| Synthesis of results | 14 | Describe the methods of handling data and combining results of studies, if done, including measures of consistency (e.g., I^2^) for each meta-analysis. | 8-9 |

*Table S1 (cont’d)*

| **Section/topic** | **#** | **Checklist item** | **Reported on page #** |
| --- | --- | --- | --- |
| Risk of bias across studies | 15 | Specify any assessment of risk of bias that may affect the cumulative evidence (e.g., publication bias, selective reporting within studies). | 9 |
| Additional analyses | 16 | Describe methods of additional analyses (e.g., sensitivity or subgroup analyses, meta-regression), if done, indicating which were pre-specified. | 8-9 |
| **RESULTS** | | |  |
| Study selection | 17 | Give numbers of studies screened, assessed for eligibility, and included in the review, with reasons for exclusions at each stage, ideally with a flow diagram. | 9, Figure 1 |
| Study characteristics | 18 | For each study, present characteristics for which data were extracted (e.g., study size, PICOS, follow-up period) and provide the citations. | 10-12, Table 1 |
| Risk of bias within studies | 19 | Present data on risk of bias of each study and, if available, any outcome level assessment (see item 12). | NA |
| Results of individual studies | 20 | For all outcomes considered (benefits or harms), present, for each study: (a) simple summary data for each intervention group (b) effect estimates and confidence intervals, ideally with a forest plot. | Table 1 |
| Synthesis of results | 21 | Present results of each meta-analysis done, including confidence intervals and measures of consistency. | 10-12, Figures 2-4, Tables 2-5, Tables S2-5 |
| Risk of bias across studies | 22 | Present results of any assessment of risk of bias across studies (see Item 15). | 13, Figures S1-3 |
| Additional analysis | 23 | Give results of additional analyses, if done (e.g., sensitivity or subgroup analyses, meta-regression [see Item 16]). | 10-12 |
| **DISCUSSION** | | |  |
| Summary of evidence | 24 | Summarize the main findings including the strength of evidence for each main outcome; consider their relevance to key groups (e.g., healthcare providers, users, and policy makers). | 13-16 |
| Limitations | 25 | Discuss limitations at study and outcome level (e.g., risk of bias), and at review-level (e.g., incomplete retrieval of identified research, reporting bias). | 16-17 |
| Conclusions | 26 | Provide a general interpretation of the results in the context of other evidence, and implications for future research. | 17 |
| **FUNDING** | | |  |
| Funding | 27 | Describe sources of funding for the systematic review and other support (e.g., supply of data); role of funders for the systematic review. | 17 |

| **Table S2: fMRI meta-analysis on EC components (attention) with age as a covariate (only contrasts without socioemotional components were included)** | | | | | | | |  |
| --- | --- | --- | --- | --- | --- | --- | --- | --- |
| Brain regions with significant peak activation | | | | | | Cluster breakdown | Network parcellation |  |
| Anatomical regions | ASD > TD/ ASD < TD | Total voxels | MNI coordinates | SDM-Z | *p (uncorrected)* | Anatomical regions (Broadmann areas) |  |  |
| **Meta-analysis of attention with age as a covariate (n = 10)** | | | | | | | |  |
|  |  |  |  |  |  |  |  |  |
| Left inferior frontal gyrus, triangular part | ASD > TD | 31 | -42,34,26 | 3.289 | <.0005 | Left middle frontal gyrus (BA45, BA46) | FPN |  |
|  |  |  |  |  |  | Left inferior frontal gyrus, triangular part (BA45, BA46) |  |  |
|  |  |  |  |  |  | Corpus callosum |  |  |
| Right precentral gyrus | ASD > TD | 15 | 34,-18,56 | 3.024 | <.005 | Right precentral gyrus (BA4, BA6) | SMN |  |
| Left cerebellum, crus I | ASD < TD | 192 | -20,-78,-30 | -3.392 | <.0005 | Left cerebellum, crus I, II, (BA18) | N/A |  |
|  |  |  |  |  |  | Left cerebellum, hemispheric lobule VI, VIIB |  |  |
| Right cerebellum, hemispheric lobule | ASD < TD | 137 | 24,-66,-20 | -3.480 | <.0005 | Right cerebellum, hemispheric lobule VI, (BA18, BA19, BA37) | N/A |  |
|  |  |  |  |  |  | Right fusiform gyrus (BA19, BA37) |  |  |
|  |  |  |  |  |  | Right inferior network, inferior longitudinal fasciculus. |  |  |
| Right cuneus cortex | ASD < TD | 133 | 18,-74,36 | -3.401 | <.0005 | Right superior occipital gyrus (BA7, BA19, BA18) | N/A |  |
|  |  |  |  |  |  | Right cuneus cortex (BA19, BA18, BA7) |  |  |
| Left middle occipital gyrus | ASD < TD | 63 | -32,-72,24 | -3.313 | <.0005 | Left middle occipital gyrus (BA19, BA39) | VN |  |
|  |  |  |  |  |  | Left inferior network, inferior longitudinal fasciculus |  |  |
|  |  |  |  |  |  | Left superior longitudinal fasciculus I |  |  |
| Left precentral gyrus | ASD < TD | 40 | -34,-16,60 | -3.279 | <.005 | Left precentral gyrus (BA4, BA6) | SMN |  |
| Left inferior occipital gyrus | ASD < TD | 40 | -42,-76,-10 | -2.998 | <.005 | Left inferior occipital gyrus (BA19, BA37) | VN |  |
|  |  |  |  |  |  | Left temporal gyrus (BA37) |  |  |
|  |  |  |  |  |  | Left fusiform gyrus (BA19) |  |  |
| Right parahippocampal gyrus | ASD < TD | 36 | 28,-34,-12 | -3.045 | <.005 | Right parahippocampal gyrus (BA20, BA37) | N/A |  |
|  |  |  |  |  |  | Right hippocampus (BA20, BA37) |  |  |
| Note: FPN = Fronto Parietal Network; SMN = Somato Motor Network; VN = Visual Network; DAN = Dorsal Attention Network; VAN = Visual Attention Netwok; DMN = Default Mode Network; N/A = not applicable | | | | | | | |  |

| **Table S3: fMRI meta-analysis on EC components (inhibitory control) with age as a covariate (only contrasts without socioemotional components were included)** | | | | | | | |  |
| --- | --- | --- | --- | --- | --- | --- | --- | --- |
| Brain regions with significant peak activation | | | | | | Cluster breakdown | Network parcellation |  |
| Anatomical region | ASD > TD/ ASD < TD | Total voxels | MNI coordinates | SDM-Z | *p (uncorrected)* | Anatomical regions (Broadmann areas) |  |  |
| Right angular gyrus | ASD < TD | 196 | 48,-72,30 | -3.333 | <.0005 | Right angular gyrus (BA19, BA39, BA7) | DAN |  |
|  |  |  |  |  |  | Right middle occipital gyrus (BA19, BA39) |  |  |
| Left anterior cingulate / paracingulate gyri | ASD < TD | 70 | -2,26,20 | -3.406 | <.0005 | Left anterior cingulate / paracingulate gyri (BA24, BA32) | DMN |  |
|  |  |  |  |  |  | Right anterior cingulate / paracingulate gyri (BA24) |  |  |
|  |  |  |  |  |  | Left median network, cingulum |  |  |
| Note: FPN = Fronto Parietal Network; SMN = Somato Motor Network; VN = Visual Network; DAN = Dorsal Attention Network; VAN = Visual Attention Netwok; DMN = Default Mode Network; N/A = not applicable | | | | | | | | |

| **Table S4: fMRI meta-analysis on EC components (cognitive flexibility) with age as a covariate (only contrasts without socioemotional components were included)** | | | | | | | |  |
| --- | --- | --- | --- | --- | --- | --- | --- | --- |
| Brain regions with significant peak activation | | | | | | Cluster breakdown | Network parcellation |  |
| Anatomical region | ASD > TD/ ASD < TD | Total voxels | MNI coordinates | SDM-Z | *p (uncorrected)* | Anatomical regions (Broadmann areas) |  |  |
| Left anterior cingulate / paracingulate gyri | ASD < TD | 388 | 0,40,16 | -3.148 | <.005 | Right and left anterior cingulate/paracingulate gyri (BA24,32) | DMN |  |
|  |  |  |  |  |  | Left superior frontal gyrus, medial (BA32) |  |  |
|  |  |  |  |  |  | Right median cingulate/paracingulate gyri (BA32) |  |  |
|  |  |  |  |  |  | Left middle frontal gyrus (BA9) |  |  |
| Note: FPN = Fronto Parietal Network; SMN = Somato Motor Network; VN = Visual Network; DAN = Dorsal Attention Network; VAN = Visual Attention Netwok; DMN = Default Mode Network; N/A = not applicable | | | | | | | | |

| **Table S5: Meta-regression of EC components with age as a regressor (only contrasts without socioemotional components were included)** | | | | | | | |  |
| --- | --- | --- | --- | --- | --- | --- | --- | --- |
| Anatomical region | Decrease/  increase in activation with increasing age | Total voxels | MNI coordinates | SDM-Z | *p (uncorrected)* | Anatomical regions (Broadmann areas) | Network parcellation |  |
| **EC component: attention** | | | | | | | |  |
| *n.s.* | | | | | | | |  |
| **EC component: inhibitory control** | | | | | | | |  |
| *n.s.* | | | | | | | |  |
| **EC component: cognitive flexibility** | | | | | | | |  |
|  |  |  |  |  |  |  |  |  |
| Left median cingulate/paracingulate gyri | Increasing activation with increasing age | 13 | -6,-38,54 | 1.705 | 0.044 | Left median cingulate / paracingulate gyri | DMN |  |
|  |  |  |  |  |  | Left precuneus |  |  |
|  |  |  |  |  |  | Left paracentral lobule |  |  |
| Left anterior cingulate / paracingulate gyri | Decreasing activation with increasing age | 93 | 0,44,4 | -2.064 | 0.019 | Left anterior cingulate / paracingulate gyri (BA10, BA32) | DMN |  |
|  |  |  |  |  |  | Right anterior cingulate / paracingulate gyri (BA10) |  |  |
|  |  |  |  |  |  | Right superior frontal gyrus, medial (BA10) |  |  |
| Note: FPN = Fronto Parietal Network; SMN = Somato Motor Network; VN = Visual Network; DAN = Dorsal Attention Network; VAN = Visual Attention Netwok; DMN = Default Mode Network; N/A = not applicable | | | | | | | |  |
